# Clinical Lectures on Some of the Principal Diseases of the Eye

**Published:** 1855-09

**Authors:** Isidor Gluck

**Affiliations:** Chief Surgeon to the Hungarian (Vilmos) Hussars, and to various Hospitals during the late war in Hungary; Cor. Fellow Med. Soc. of London, etc.


### BUFFALO MEDICAL JOURNAL

AND

### MONTHLY REVIEW.

**VOL. 11.** SEPTEMBER, 1855. **NO. 4.**

### ORIGINAL COMMUNICATIONS.

ART. I.—*Clinical Lectures on some of the Principal Diseases of the Eye.* Delivered before the Class of the New York Medical College. By ISIDOR GLUCK, M. D., Chief Surgeon to the Hungarian (Vilmos) Hussars, and to various Hospitals during the late war in Hungary; Cor. Fellow Med. Soc. of London, etc.

### ON OPACITIES OF THE CORNEA.

GENTLEMEN,—The disturbance of sight complained of by this individual, is attributable to the result of a series of organic destructions caused by inflammation and its consequences; the function of the organ of light being materially dependent on its normal anatomical construction, will be greatly impeded by any change contributing to alter, if not destroying, altogether, the textures constituting the eye.

The morbid change in the cornea is apparent to you even from a distance, while the patient himself is annoyed by its effect, causing, as he asserts it does, trouble of sight. With both eyes used together the young man sees misty; it does not require, however, great acuteness to recognize that the opacity in the left eye, although twice as large as that in the *right one*, being situated lower than the pupil, does not cause the dimness, but that the opacity in the right eye alone being situated a little in front of the pupil, creates a disturbance of sight. Thus by looking with the left eye objects appear to him clear, whereas they appear nebulous if looked at by the right one, or by both eyes.

Before entering into the *pathological* conditions of the cornea, it will be necessary to recall to your minds its *normal* state. While demonstrating the anatomy of the eye, I remarked to you, gentlemen, that the cornea is constituted of the *epithelium*, situated in layers, consisting of nucleated cells, and of the *proper* corneal substance, which is a lamellated fibrous texture.

The epithelium of the cornea connects on the border of the cornea with the epithelium of the limbus conjunctivalis, which is a continuation of the fibres of the connecting tissue forming the conjunctiva, and is rich of vessels. The proper substance of the cornea consists of a great number of lamellæ,* situated like concentric layers, one upon another, and resembling each other perfectly in their structure and physical properties. They are composed of very thin and transparent fibres, which parallel in every layer, but differently in various ones.

The fibres are kept together by a gelatinous, textureless hyaline, very hygroscopic mass, which fills out the interlamellar spaces of the cornea. The basis of the cornea causes, by its property of attracting fluids, its swelling when soaked in fluids and the succulence peculiar to the cornea which is so great that fresh eyes allow their contents to escape on the surface, by a moderate pressure, in the form of drops, is mainly attributable to the basis of the cornea.

To it is also attributable the *optical equality* and the great transparency of the corneal parenchyma, filling out, as it does, the interspaces between the fibres, and causing also the resistance of the cornea. The cornea becomes, therefore, opaque and atonic, if its succulence is diminished by evaporation or expression of the contained fluid; but the corneal lamellæ do not extend to the border of the selerotic coat, for between both the cornea and the selerotic exists a stratum of a tough, firm, transparent, entirely homogeneous, perfectly textureless substance, which by the naked eye cannot be distinguished

* The mechanics have long ago valued the resistance of lamellated walls and construct, therefore, the walls of such parts of the machine as are exposed to a higher pressure of lamellæ ; thus, for instance, of a *steam boiler*. If the cornea was woven felt-like, it could not retain its form; so in woven textures the single fibres run too long to be able to resist, sufficiently, a high pressure acting on their course under a great angle. The lamellated structure of the cornea causes, by an intense force acting on the eye by means of an obtuse instrument, rather a recession of the iris from the ciliary muscle, or a breaking up the selerotic coat, than that of the cornea.

at all from the lamellated cornea; this *intercalar* substance forms a border of the thickness of the cornea and is $\frac{25}{100}'''$ to $\frac{5}{100}'''$, wide. It is easily perceptible in a morbid cornea, being deprived of any organization; existing on the lowest degree of capacity of animal formation, it does not participate in the pathological processes of the lamellated corneal substance and thus confines, externally, distinctly the diseases. In this stiuctureless marginal substance the lamellæ of the cornea loose themselves without a defined border, their periphery disappears in the homogeneous intercalar mass; on the other hand the selerotical fibres enter under various angles the border of the selerotic coat. In its texture the cornea contains marrowless, pallid nerve fibres, belonging partly to the superior cervical ganglion of the sympathetic nerve, partly to the ciliary ganglion and to the naso-ciliar branch of the trigeminus; they run as ciliary nerves between the choroid and selerotic coats to the ciliary muscle, and only then separating from the accompanying nerves enter again into the selerotic coat in order to pass, connected in 12 or 28 small trunks through the border of the selerotic coat and the textureless marginal substance of the cornea, to its interlamellar spaces, where they divide dichotomously in more and more subtile branches, and thus form an irregular net with large loops and extremely fine anastomoses, the trunks of which have mostly a centripetal direction.

The sensibility of the cornea is not very great, on account of the small quantity of the sensitive nerves, and of their small capacity of conducting in a centripetal direction; in the morbid state, however, the sensibility of the cornea is increased, although it may be almost deprived of any sensibility when normal, and thus the highest degree of pain may be excited in it. The cornea not only serves as a protecting membrane to the eye, but at the same time acts as a link of the dioptic apparatus. As such it has to possess a certain degree of firmness, having to support on its posterior wall the pressure of the contents of the eye from within, and to oppose the necessary resistance. On the other hand it has to resist the mechanical forces from without and thus to sustain its light-refracting walls in an adequate situation without being brittle and endangered by somewhat sudden blows or pressures. This is realized by the lamellated structure of the crossing fibres situated over each other. This arrangement prevents an extension of the curvature of the cornea, while it admits during blows and pressures on its surface the necessary curvable elasticity.

The extreme smoothness of both surfaces of the cornea and the optical quality of the corneal substance, cause the very small diffusion of the light falling on it, *i. e.* the *slight visibility of the cornea.* This is an important means of diagnosticating diseases of the cornea; it will be the more visible the less its transparency, in consequence of the change of the *optically equal* texture of the cornea. Thus abrasions of the corneal epithelium and transparent exudations may be diagnosticated by the increased diffusion of light produced by them. For this reason you see *here* the opacity in both eyes without difficulty. The increased diffusion of light produced by the transparent part of the cornea shows a change of the corneal texture, and even in the opacity itself you observe differences of shade of light and color, from the fact that the optical equality is disturbed variously through the difference of the altered texture showing the opacity.

The texture of every organ is the result of a peculiar formation out of the plasma necessary to it, which latter although *formally* for all organs *homogeneous*, still chemically must be regarded as a *specific different* one. The capacity of formation peculiar to each organ, may be considered as a part of the vital energy common to the whole organism. The abnormalisms of texture may be looked upon as produced by changes of the plasma contributing to the *growth* of the organ, or as the result of an altered specific vital energy, modifying the process of formation. We will confine ourselves, however, to such alterations of the cornea as are *objectively* perceptible or visible. The loss of optical equality depends upon the existence of anomalous elements entering into the formation of the parenchyma of the cornea or of its epithelium. Let us follow the formation of the cornea and observe it when in a pathological condition. Inflammation in the cornea is commonly regarded as the most frequent cause of alterations, and hence resulting opacities. The cornea proper being, as above remarked, in its normal condition entirely destitute of vessels, an inflammation of its parenchyma is almost inadmissible. Although the formation of vessels during inflammation might induce the belief in the preëxistence of such vessels, which being of a small calibre, admit but serum, and in their morbid condition subsequently filled, may carry red corpuscles of blood, and thus are more apparent; still it must be admitted that inflammation of the cornea exists also *without* the formation of vessels, and moreover, their presence is not always proportionate to the exudation.

Sometimes the greatest development of the vessels is accompanied by comparatively small exudations, whereas in other instances the profusest exudations forming in cancer and tubercle of the cornea, and all such exudations which put on a genuine fibrinous, firm, and tough product, assuming a higher organization by splitting into fibres or remaining in the crude state, primarily run through their course without even the slightest vestige of a formation of any vessel. The microscope, however, gives an additional proof of the independence of the exudations in the cornea (in keratitis) from bloodvessels and stagnation, and thus shows eminently the difference of the inflammation in the cornea from that of any other organ. The blood corpuscles occurring in fresh inflammatory centres, show by physical properties their youth, and differ in every respect from those circulating in the rest of the organism. They appear very pale, commonly yellowish, of an irregular round shape, of very different sizes; are very soft, and possess almost homogeneous contents, divided only in a very small number of dark granules. The lymph around it is prevalent, and keeps them aloof from each other, rarely are visible any of them connected, they are mostly loose, enclosed in entirely irregular interspaces or loops of the infiltrated corneal texture, which loops in the beginning are irregular and radiating in branches, later, however extending in one or another direction, and branching off in channels which communicate with blood channels in the vicinity, and at last also by their intermediation with the vessels of the general circulation. But always are the walls of the vessels as yet wanting, and thus they differ from the capillary vessels by their greater diameter, which is unequal in the different parts of their length.

In the injections of the inflamed portions of the cornea are visible, evidently, newly formed interspaces for receiving blood, in which every vestige of stagnation is wanted, as neither an agglutination of blood corpuscles is observable, nor an agglomeration of inspissated, deep colored coagulated fibrin, or solidified albumen, is visible; nor do appear within those channels the phenomena of the formation of nuclei or cells foreign to blood. It remains, therefore, but to assume that the new formation of elements cannot be derived from an immediate exudation through the walls of vessels, but that the existence of elements foreign to the normal structure of the cornea, is attributable on the one hand, to the aqueous humor which pervades and nourishes the cornea; on the other hand, to the alteration of the specific vital energy proper to the cornea, which latter cause by itself, mostly produces the development of anomalous processes of formation in the cornea, to which it is confined, and therefore, may be considered a *local* morbid process. It cannot, however, be denied, that often the principal and only cause will be in the constitution of the blood itself; at the same time it must be admitted that often the character of the deposit will be at variance with the constitution of the blood. Thus it may and does happen, that in an evidently strumous individual, one tainted with cancerous, tuberculous, or a lupous dyscrasia, an exudation in the cornea, or rather deposit on or in it may take place of a genuine fibrinous character, or of a deposit capable of splitting into fibres.

You may infer from the mentioned circumstances that the appearance of certain casual forms of the exudation of certain colors and consistency, is not sufficient to establish a diagnosis.

As the division of the exudation, according to its chemical nature, is utterly impossible, unless an analysis is instituted, it may not, perhaps, be improper to classify it according to Stelbwag, into

*A.* Such as are not capable of formation, or transformation, but remain in the crude state.

*B.* Into such as are capable of transformation.

*A.* The exudation may not possess the property of being transformed into other protein compounds, but it retains its albuminous nature, and remains in the very same state in which it has been deposited in the parenchyma of the cornea, or its epithelium (unaltered) and at the most alters its state of aggregation by solidifying in consequence of alteration of its finest particles, a process not based on the principle of vitality.

The appearance of such a product is manifested by *changes in the transparency of the cornea.* The cornea assumes, in a smaller or larger extent, often in its whole circumference, a peculiar jellylike appearance; it appears dull, greyish, with a tint of blue or yellow, without being changed in thickness or density.

Often this is the only symptom; more often, however, its surface loses the brilliancy, and looked at in a certain direction opalescence is observed in it. Seen closely the cornea appears rough, as if full of small excavations, as if pricked with needles in its whole extent or in some of its parts. The disease may exist for a long time in this stage, and the reparative process may develop itself, carrying off every product without leaving any vestige. Usually, however, in the homogeneous cornea, roundish aggregations form themselves, of small patches the size of poppy seed, of a dull grey color, with a yellowish or bluish tint. They are situated in various depths of the cornea, as is easily observable when the cornea is looked at sideways. They cover each other in some parts and appear to the naked eye confluent; they are most crowded in the posterior lamellæ of the cornea, where they really *lose themselves* in more or less extended nebulous opacities.

This opacity of the cornea is caused by the formation and appearance of an extremely fine, light, dust-like molecular mass, in the fibrinogenous base (substance) which pervades the fibrous structure and the epithelium of the cornea. The structure of the corneal lamellas is unchanged, but appears as if smoky, opaque, and changed in hue. The contents of the epithelial cells appear also dim, dull, without any accumulation of granules in a larger than the normal quantity. In the more opaque patches only, the molecular mass seems to have densified itself in consequence of reciprocal attractions of the molecular mass, the granuli appear there of the most different size and form, of a very dark color, and grouped together in a nebulous dark granulous base substance, but always without indication of a higher organized element of formation.

With the process causing opacity or dimness in the half fluid base of the corneal texture, seems also to suffer the process of vegetation of the epithelial cells, and separation of them follows, as the roughness of the cornea is produced by the loss of single epithelial cells on various spots of the surface of the cornea.

*B.* Exudation capable of organization.

Its characteristic property is the capability of being organized in elements of texture. The metamorphoses and phases in which the product appears, being various, the form under which it appears is accordingly modified and appears differently. The metamorphosis, however, always takes place in a certain way, the elements of form develop themselves constantly out of jelly-like fluid, homogeneous in all instances, a more or less succulent mass, which pervades, more or less, the corneal texture, and thus deprives it of its smoothness and transparency, and appears under the microscope as a viscid structureless substance, which is characterized by the great number of fine, light, dust-like molecules. This is, according to Stelbwag, the *base form* of each corneal exudation.

Keratitis, or inflammation of the cornea, may remain unaltered in its *primitive* form; usually, however, the exudation undergoes metamorphoses which may be reduced

(*a.*) To simple splitting in fibres of the coagulating product, (keratitis simplex.)

(*b.*) The metamorphosis may consist in the formation of vessels with a contemporaneous formation of epithelium and a fibrous texture, (keratitis vasculosa.)

(*c.*) The exudation is partly fluid, partly plastic, forms vessels; the process governing the metamorphosis is a typic one, and shows eminently to be dependent upon the alteration of single sensitive nerve tubules, (herpes cornealis.)

(*d.*) The exudation is transformed, or partly so, to a pus-like fluid, (keratitis suppurativa.)

(*e*.) The exudation enters the carcinomatous metamorphosis, (keratitis carcinomatosa.)

(*f*.) The exudation may be a lupose product, (kerat. luposa.)

Ructe assuming that the inflammation is* a process based on an altered nutrition, in which everything belonging to the nutrition of a part is interested, (the blood and nerves as well as the walls of the vessels and texture) supposed that the inflammation may originate in every part contributing to nutrition, thus, in the blood, nerves, vessels and textures, and thus subsequently concern or disturb the normal relation of the parts. Presuming further the correctness of the existence of the vessels recently demonstrated in the normal cornea by Caccius, he divides a keratitis.

(*a*.) Nervosa.

(*b*.) Vasculosa.

(*c*.) Parenchymatosa.

The inflammation of the cornea in general, may terminate in

1. Resolution.

2. Enlargement, or rather new formation of bloodvessels.

3. Mortification of the cornea.

(*a*.) Gangrene.

(*b*.) Keratomalacia.

(*c*.) Ulcera corneæ.

4. Keratocele (hernia corneæ) prolapsus iridis, and staphyloma corneæ racemosima.

5. Abscessus corneæ.

6. Opacities of the cornea.

With this latter termination of the keratitis, we meet in this young man, who, as you hear, has suffered already when a child from a violent inflammation in both eyes, and under the treatment of the late Dr. Rogers improved, when a few years ago, a violent inflammation of his eyes, in consequence of a cold, nearly destroyed his sight entirely, Slowly recovering from this affection that lasted about three years, he regained his sight, but dimness and imperfect vision compelled him to apply here. The opacities in both eyes did not diminish, but on the contrary, extended after the second attack. In this instance an iritis has in both eyes established a connection of both irides with the anterior capsule of the lens, which connection prevents the enlargement of the pupils, although atropine has been used for that purpose. The brownish-red color irregularly deposited in form of patches, and

*According to Virchow.

contrasting with the original grey color of the irides, are, in both eyes, more distinct near the pupillary margin where the exudation was more profuse, on account of the anatomical construction, than in the rest of the irides. The oval shape of both pupils very little, or at all altering by the different degrees of light, permits the perception of the objects clearly in the left eye. The extent of the pupil does not exceed 1‴ in length and ½‴ in breadth, in the left, and ¾‴ in the right eye. It would be presumptuous to decide whether the iritis followed or preceded the corneitis in the different attacks, but it may be inferred by the visible remnant of an ulcer in the cornea now appearing as a cicatrix in the left eye, that the connection of the iris with the lens (synechia posterior) may have taken place already before such an ulcer was established, and thus prevented the union of the iris with the cornea, (synechia anterior.)

It would lead me too far to enter now into the other forms of keratitis than that to which, in this instance, the opacity is attributable; but the general outline of the appearance, and possible metamorphoses of such products as cause the opacity, together with the manner in which they alter the structure, and consequently the function of the eye, it will be necessary to dwell upon.

Undeniable is the result, although not accountable with absolute accuracy, whether mainly attributable to an endo and exosmotic process, or to that intermediated by vessels whether preëxisting or newly formed during the morbid process, or whether by both means an inflammation of the cornea has established its product—enough, the visible altered structure of the cornea is the consequence of it, and causes the disturbance of sight. I do not presume to say that it is immaterial respecting the treatment, to know in what way the product of inflammation has been formed; but the one and the other theory, based partly on facts, partly on speculative induction, has so many arguments in its favor that the possibility of both principles must be admitted, but till now it could not be ascertained exactly under what circumstancs the one or the other, or both together, take place. Stelbwag's careful anatomico-pathological examination, controlled by the appearances in the living diseased eye have by his regard to the physiological processes in it, the greatest claim to attention, and his inferences drawn from and formed on the positive ground of dissection, deserve closer consideration.

The different formation of the elements deciding the form, appear as transitions or links in the chain of the morbid process, by which means the morbid product becomes capable of being resorbed or separated.

Without entering further into the formation of the corneal exudations as products on the way of being metamorphosed, and without following the changes of the different elements of form in their various stages of evolution or retrogression, I will confine myself, to-day, to the consideration of those products which attain a degree of development, on which, although subject to a continual change of element, the same alter *little*, or *not at all*, their external form and texture, *i. e.*, the products remain *stationary*. Such products are epithelium, loose connecting tissue, fibrous lamellated cicatrix-texture ossifications, cretaceous and fatty formations. Rarely occurs one or the other new formation by itself; usually they are variously combined and mixed with each other in the most different quantities.

### EPITHELIAL OPACITIES.

Anomalous epithelial layers produced by morbid processes of vegetation, remain often, without changing materially, when the pathological process is terminated.

Only superficial layers of exudation transform themselves to epithelial textures. Morbid products included in interstices of the cornea never transform into elements proper to the epithelium. Morbid, as well as healthy, epithelium, is constantly exposed to a change of its textures of form, the oldest most external layers *separate* constantly, while out of the interior matrix new blastema is formed, out of which cells are produced which, in their continual evolution, are gradually placed forward by the subsequent layers, and arriving at the surface desquamate themselves. The continued formation of morbid opaque epithelium on the convexity of the cornea, depends upon its morbid substratum, which if morbid will give rise to a formation of morbid epithelium, as shown by the microscope. The aqueous humor which serves in the normal condition as nourishment to the cornea, and partly is transformed to epithelium on the surface of the cornea, may be metamorphosed during its transit through its morbidly changed cornea, or the formation of transparent epithelium may be prevented through the metamorphosis of the cells *not* in contact with the normal parenchyma of the cornea.

A corneal exudation may become stationary, but it depends much upon the quality and quantity of the exudation, its greater or less tendency to a higher organization, etc. If the exudated layer is a very thin one, it comes to a formation of a superficial epithelial layer, whatever the constitution of the exuded mass may be, its new formation amounts to epithelium with a subjacent structureless opaque mass. In the profuse exudation, on the contrary, it depends upon the constitution of the new product what textures of form can be developed of it. Genuine fibrinous massy products almost always metamorphose themselves to a tough tendonous new product, adhered only to a very thin layer of epithelium.

Profuse fibro-albuminous exudations, which show already during inflammation a great tendency to form cells and appear as pannus or ulorous granulations, transform themselves into stationary new formations, which mostly consist of loose connecting tissue, covered with numerous epithelial layers and subjacent rigid tendonlike texture; or they are almost entirely composed of epidermislike epithelium, which seems to be separated from the foremost layer of the healthy corneal parenchyma through a structureless, or at the most indistinct fibro-striped, very tough, firm, dry, and cartilagelike substance, which often shows its small capacity of development by the blending of its deeper layers with lime and fat crystal conglomerates. Epithelial new formations of the cornea, of inconsiderable thickness, are simple haziness or dullness, simple patches, cloudy or smoky. You need no better proof as to how suddenly a cornea may get alternately obscure and cleared, than the case of the French girl you saw here frequently applying, and exhibiting a keratitis in its different stages, and with exudations on, in, and under the corneal epithelium.

### OPACITIES IN THE PARENCHYMA OF THE CORNEA

Are of two forms, materially differing from each other. Opacities resulting from changes in the corneal substance itself with or without formation of ulcers, (primary or secondary,) are the most frequent. They come under various names: leucoma, margarite or perla, albugo or paralampsis, cicatrix. Exudations between the yet existing lamellæ of the cornea, and exudations compensating the lamellæ lost in consequence of an ulcerous process, or of mechanical or chemical injuries, occur together; in fact the latter one never occurs without the first in the circumference of the cicatrix. The opacities may be caused

(*a.*) By the deposit of fibro-albuminous exudations between the more or less *unchanged* corneal lamellæ.

(*b.*) By deposit compensating the corneal substance lost by ulceration or destroyed by escharotics, and may be partly capable of transformation into a *proper* corneal substance, partly incapable for it, but remains unchanged as fibrous and cicatrix texture, or it may appear as mostly and usually *stationary* in the center of the opacity, and capable of transformation in its periphery.

### CATOPTRIC RELATIONS.

However differently their texture may be composed, they unite in one principal property, characteristic of every such neoplasy, namely in *the want of optical equality in its texture and in the quality based upon this property of diffusing, regularly, the rays of light falling on it.* Each element of new formation (neoplasic) may be considered as a source of light from which emanates a spherical wave of light which is *excited* partly by light diffused in the world, partly by that reflected from external objects and turned on the neoplasic formation. One part of this spherical wave, equally progressing in all directions, diffuses itself in the *outer world*, and intermediates as the so called *reflected* part, the *visibility* of the new formation (neoplasic), as dim, or according to the constitution of the single forms of element, variously colored patches. The absolute quantity of light, coming from the new formation in a certain direction, will be the greater, and the patch will appear the more intensely colored, the greater its illumination, and the more the elements of new formation diffuse the light, the more *optically unequal* its texture is. Therefore appear, under the same intensity of illumination, calcareous or chalky exudations of a more saturated color than the epithelial new formations do, and the latter ones more saturated than loose connecting tissue (if it is not very rich of vessels) and the fibrous lamellated cicatrix.

A section of each wave excited on the surface of the new formation, advances in the same direction with the direct transmitted light. If it meets in its way with a new stratum of optically unequal texture, the process that took place on the surface repeats itself, and so on in each stratum a new part of the coming light is reflected, and thus the visibility of the cornea increased. Its absolute light increases, not only with the intensity of illuminations and the degree of optical unequality, but also with the thickness of new formation.

Very thin strata of a new formation reflect but little light, their image is projected on the dark back ground and the translucent iris; they appear grey or bluish, but thicker neoplasies (new formations) appear in the color proper to their elements.

The quantity of the transmitted light is in inverted relation to that reflected; the light, therefore, emanating, or reflected, from some object and falling on the cornea, will be the less transmitted, the *thicker*, the *more* optically unequal the newly formed texture is, and the less the illuminating intensity of an element of the surface.

Therefore if a new formation occupies the whole of the cornea, or darkens that part of it situated opposite the pupil, it follows that by the same illuminating intensity of the opaque corneal space, the rays coming from an external object, will be the less transmitted to the retina (the object will be the less visible) the less optically equal and the thicker the new formation is.

In every instance of an extended opacity on the cornea, or of that part corresponding to the pupil, strongly illuminated shining objects will be seen better than less illuminated and dull ones, larger ones better than smaller ones, nearer objects better than distant ones, and such ones as are situated in the optical axis will be perceived better than objects placed sideways.

And as further the apparent brightness of a retinal image *decreases ceteris paribus*, the larger its extent in space, and this latter increases the more, the more distant out of the range of accommodation, proper to the eye, the object is situated, it is evident that the perception of an external object through an opaque cornea becomes the more difficult, the less the dioptic apparatus is capable of adapting itself to a corresponding appropriate distance. Very remote objects, situated far beyond the distant point of vision of the eye concerned, will always be seen *duller* and *more* imperfect than even less intensively illuminated and relatively smaller ones, if they are within the range of vision. It is further apparent that neoplasmas, if connected with flatness or diminution of the radius of curvature of the cornea will impede the sight, more than new formations which exist without change of the normal curvature of the cornea, as in the former case the divergence of the united transmitted rays will fall the more in front of or backwards the retina, the greater the abnormalism of the curvature of the cornea. Eyes with a flatened or cicatrised contracted cornea, or with a cornea that protrudes staphylomatously, see always worse than such eyes as have retained the normal curvature of the cornea, even if the cornea is more opaque than in the former instance.

Corneal opacities act on the retina, under certain circumstances, like illuminating objects, and such opacities are translucent, transparent. One part of their light is reflected, and another transmitted; part is diffused on the retina; the diffused rays disturb the formed retinal image if the proportionately much larger quantity of the regularly refracted light does not compensate the disadvantage produced by the diffused light.

The diffused rays of light, if intense, cover the regularly formed retinal image with a fog, hence the retinal image is not clear, so much so that the color, clearness, and the curvatures of every object appear indistinct.

Translucent central specks impede the perceptions of objects more than eccentric ones, because in the former the light which not forming the retinal image appears nebulous, falls on the center of the retina, whilst the eccentric opacities send their light to a corresponding eccentric part of the retina, and admit only a small quantity on its center. The diffused rays of light coming from translucent opacities, are therefore the more intense the larger the speck, and the more intense the rays of light. For that reason patients, with transparent opacities, see best high colored objects in a brown fog, or by a dull illumination and subdued light; and on the contrary, dull objects best in a stronger light, with white fog or mist.

This patient, you will now understand, is prevented from seeing distinctly with the right eye through the *translucent* opacity covering the lower part of the pupil, and extending somewhat below it. The adhesion of the iris with the anterior capsule, is here of great service, as the diffusion of the rays coming from the half-transparent opacity would be greater, and thus the disturbance also, if an enlargement of the pupil was possible; but fortunately for the patient the iris excludes the entrance of the diffused rays of light, and thus nature endeavored to remedy, in some measure, the evil. In the left eye, where the opacity is entirely below the pupil, which is also incapable of enlargement, the sight is not disturbed.

Although the opacity in the left eye is one by appearance totally different from that in the right, they still seem to have originated with the same process.

The impediment of vision produced by new formations situated eccentrically on the cornea, depends upon the distance of the central border of the new formation from the optical center of the cornea, further upon the diameter of the pupil, and upon the temporary state of accommodation of the eye. The pencil of light coming from the translucent opacity, situated eccentrically, will fall the more outside the macula lutea, the more distant the central margin of the opacity is from the optical axis. The contraction of the pupil must be the greater the nearer the central border of the transparent opacity to the optical axis in order to exclude the diffused rays of light from the center of the macula lutea. If the diameter of the pupil is insufficient, the accommodation of the eye to near objects has to compensate it, by giving such a form to the lens, or according to believers in locomotion of the lens, to move it forward, as by it the angle in which the axis of the cones of light coming from the new formation cut the optical axis, increases. The shortening of the distance of accommodation is generally combined with diminution of the pupil, which process explains why patients, with eccentric corneal opacities, see *near* objects clearer and more defined.

### INTRANSPARENT PATCHES OF THE CORNEA.

In the normal condition of the eye where the rays of light coming through the cornea from a luminating point form a cone, the base of which is situated on the cornea, and the apex of it on the object, the patient does *not* observe patches smaller than his own pupil, as from each cone of light rays enter yet on the sides of the opacity sufficient in quantity to form, by an adapted accommodation, a clearly defined retinal image. This is only proportionately darker, such an opacity cannot throw a shadow on the retina, *i. e.*, make a part of the object invisible. The base of the cone of light falling on the cornea and necessary for the visibility of the object, is somewhat larger than the pupil, because the rays begin already to converge at the cornea.

A dark macula, in the center of the cornea, causes blindness in the brilliant light, on account of the contraction of the pupil; in subdued light, however, having the size of the base of the cone of light, of a larger diameter than that of a medium sized pupil, or after the use of a nyctiopticum the patient may see sideways the opacity.

Two cases of corneal opacities now under my private treatment, will illustrate both the corneal morbidities and the phenomena observable hereby. I have taken particular pains (on account of the peculiarity of the case) of gaining the permission of introducing to you one of them. This young lady of Morrisania, 14 years of age, when four years and a half old, fell on her left side, bruising the head on the same side; twelve months later an inflammation, whether or not in connection with the injury I am not prepared to say, established itself in the left eye, and shortly after a violent inflammation in the right one, appeared on the sufferer, until about six years ago an operation on the right, performed in Dublin, resulted in total blindness. Now the young patient cannot even distinguish light from darkness by it, not only the opacity of the cornea, but the entire disorganization of the eye that evidently shows results of a chronic choroidal inflammation, prevents the perception of light. The left eye, that particularly deserves our attention, is covered in the center of the cornea with a large bluish-white speck, covering entirely the pupil, and a large portion all around the iris. The patient, as you observe, is blinded, can neither walk alone nor recognize the features of any of you, neither turned toward or from the light. But an instinctive assistance of the patient will allow the imperfect perception of large objects if situated close. You observe that the left hand, cylindrically closed, is p essed by the patient against the left *malar* bone, while the thumb pushes the skin and muscles of the face upward, the index draws the upper eyelid close to the inferior one, thus forming, by the contemporaneous contraction of the orbicular muscle, a small aperture for the entrance of the rays of light in one particular spot of the cornea, excluding by it the rays falling under other circumstances on the opacity, and causing a diffusion of light, preventing the formation of the perception of the retinal image. The great inconvenience of such an exertion, aggravated by the swelling often recurring in the cheek from the repeated pressures on it, in order to accommodate and adjust the eye, is apparent to you, and is the chief and sufficient reason for her desire of regaining the capability of seeing. The exudation in the cornea is situated partly between the epithelium and the corneal lamella, partly between the lamellæ themselves, which in the center may be substituted by a morbid tissue, but the difference of its several portions is evident; whereas the central part of the opacity is greyish-white, its marginal portion, chiefly the upper border, looks washed off, losing itself gradually in the healthy normal structure. However little hope there may be for an absorption of the central parts of the opacity, it may be allowed to infer, by the appearance of the border, that a resorption in it might contribute much to the clearness of the cornea. Extended as the half transparent opacity is, it admits the entrance of light more in the upper than lower part of the cornea, where the opacity is apparently thicker and larger.

*Treatment.*—A great number of stimulants and astringents have been recommended and used for the removal of opacities, and for restoring the transparency of the cornea: Extr. cicutæ; chelidonii; argent. nitr.; subl. corros; cadmium. sulph.; sal amm.; sacch.; borax; baryt. mur.; kali caust.; sal vol. corn. cer.; merc. precip. rub.; ung. citr.; kali hydrg., and powders soluble and insoluble. The most, if not all those powders, act mechanically, producing an irritation and inflammation. Electricity and acupuncture has also been recommended. Remedies, internally applied, have been combined with the local treatment.

The removal of that portion of the cornea in which the opacity resides, was known in the time of Galen, but the practice has been allowed to fall into oblivion; but later it was revived and used, with various modifications, by Mead, Larry, Wardrop, Darwin, etc.; and still later, on suggesti n of Dr. Gulz, (Vienna) French Surgeons, chiefly Malgaigne, practiced it. Dieffenbach even resorted to the bold removal of a leucoma from the center of the cornea and brought together the edges of the incision by suture. Earthy deposits have been removed from the cornea in two instances, by Mr. Goodman; one case was under treatment of Mr. Dixon.

In all those instances the removal of the opacity was effected with the view of thinning the cornea, and thus to make it permeable to light.

Prof. Danders has lately devised "stenopic spectacles," based on the principle of excluding the rays of light from the opacity, and admitting their entrance through a small opening in a glass, or any other suitable material held close to the eye and darkened on its posterior wall, thus to admit behind it the greatest possible enlargement of the pupil. I will revert to their construction and use on another occasion. However useful they may be, and in many instances really are so, (and save the trouble and inconvenience of an artificial pupil) still to dispense with them if possible, and see conveniently without them, is certainly the greatest desire of every patient who has to use them, the more so as the stenopic* spectacles are little calculated to *improve* the appearance of the patient.

From what I have stated to you, you will understand that in half transparent opacities it is important that the quantity of light necessary for the image should exceed the quantity of diffused rays, preventing the formation or perception of the image: the larger the transparent space, the greater the possibility of entrance of light through it, provided the pupil is capable of enlargement. Holding, in view this fact, the removal of the peripherical part of the opacity would, in many instances, be sufficient to restore sight, and is, for many reasons, preferable to the formation of an artificial pupil. With this view I propose to perform an operation, for removing the opacity, in the following manner:

In young individuals, where the inflammatory process is a very active one, chiefly if the eye has, as in this instance, been repeatedly the seat of violent inflammations, every irritation extending over the eye may be followed by serious consequences, I should abstain for that reason from using such remidies as above mentioned, the more so as many, if not the most of them, have been used by the different medical men under whose care the young lady has been previously.

Considering that nature often intends to renew and repair the loss of substance (and if possible by such material as is more or less prepared and adapted for it,) I propose to remove the epithelium covering the center of the opacity, thus to induce a reparative process to resorb the exudation in the periphery

* *Stenos*, narrow ; *ope*, window glass.

of the opacity, which evidently looks capable of metamorphosis, and to convert it as substitute for the lost epithelium.

I shall confine myself, in this instance, to the center of the opacity, whereas in the young man I will remove the epithelium in that part of the opacity situated *below* the pupil, not to produce an inflammatory action in the part opposite the pupil. I will proceed to effect it at once in the lad. I use for this purpose Gimbernat's speculum, consisting of a ring slit on one side and provided with a semicircle in its upper part, which by being pressed toward the ridge on the palpebra superior of the orbit, forms with the ring calculated for steadying the eye and the handle, an ophthalmostate, which by pressure toward the eye keeps the cornea expanded and stretched. With a cataract needle curved on its blade, I now remove by its edge, holding the handle horizontally scraping in single strokes the *epithelium and some subjacent lamella.* You perceive now an excavation; we will allow nature time to fill up this loss and repeat the same manœuvre as soon as it appears replaced; at the same time ice water must be used to check a great amount of subsequent inflammation, and thus to control it. The same operation I intend to execute, to-morrow, on the eye of our obliging young lady.

(To be continued.)

